# Augmented reality with intraoperative indocyanine green lymphatic mapping in colorectal cancer: personalized surgery or a glowing distraction? A scoping review

**DOI:** 10.20452/wiitm.2025.17980

**Published:** 2025-09-09

**Authors:** Solomiia Semeniv, Michał Pędziwiatr, Justyna Rymarowicz, Mateusz Rubinkiewicz

**Affiliations:** Department of General, Oncologic, Metabolic, and Emergency Surgery, University Hospital in Krakow, Kraków, Poland; Center for Innovative Medical Education, Jagiellonian University Medical College, Kraków, Poland; Second Department of General Surgery, Jagiellonian University Medical College, Kraków, Poland

**Keywords:** colorectal cancer surgery, fluorescence imaging, indocyanine green, lymphatic mapping

## Abstract

**INTRODUCTION:**

Colorectal cancer (CRC) is a leading cause of cancer-related death globally, where precise lymph node (LN) assessment remains critical for accurate staging and prognosis. Indocyanine green fluorescence imaging (ICG-FI) has emerged as a potential tool to enhance intraoperative lymphatic visualization and guide tailored lymphadenectomy.

**AIM:**

This scoping review evaluated the current evidence on ICG-FI lymphatic mapping in CRC surgery, focusing on its impact on surgical outcomes and identifying research gaps.

**MATERIALS AND METHODS:**

A comprehensive literature search of the MEDLINE database (2005–2025) was performed using the Preferred Reporting Items for Systematic reviews and Meta-Analyses extension for Scoping Reviews guidelines. The included studies investigated ICG-FI lymphatic mapping in adult CRC patients. A qualitative synthesis was conducted across the following thematic domains: mesenteric mapping, sentinel LN (SLN) assessment, and lateral pelvic LN dissection (LPLND).

**RESULT:**

Of the 67 records identified, 34 studies met the inclusion criteria. Several studies demonstrated ICG-FI safety and feasibility, with high lymphatic flow visualization rates (75.4%–100%) and improved LN yield. Aberrant LN detection occurred in up to 50% of the cases, although these were rarely metastatic. SLN mapping showed high detection rates but variable sensitivity (63%–75%) and frequent false negatives. LPLND guided by ICG-FI showed a potential in reducing lateral recurrence, but not in improving overall survival.

**CONCLUSION:**

ICG-FI enhances anatomical precision during CRC surgery and facilitates individualized lymphadenectomy. However, its oncologic benefit remains unproven. Standardization of protocols and further prospective studies are required to validate its clinical utility and long-term impact on patient outcomes.

## INTRODUCTION

Colorectal cancer (CRC) is a malignant proliferation of abnormal cells in the colon or rectum mucosa, ranking as the third most prevalent cancer globally. With a 10% incidence among all cancers and high mortality rates, CRC remains a significant public health challenge as the second leading cause of cancer-related deaths worldwide^1^ Lymph node (LN) assessment for metastasis in CRC is a key prognostic factor, strongly linked to a higher recurrence risk and reduced survival rates, requiring precise staging to guide adjuvant therapy.^2^ Appropriate LN dissection is considered crucial for long-term survival, and international guidelines recommend evaluating at least 12 nodes to ensure accurate staging and optimal outcomes^3^;^4^;^5^

Traditionally, metastatic spread has been understood as a stepwise process, involving an orderly progression from the primary tumor to regional LNs along the lymphatic pathways that accompany the arterial blood supply. Consequently, the standard extent of resection, which includes the origins of the arterial vessels, should effectively remove all potentially involved LN stations.^6^ However, recent data suggest that this traditional paradigm and standard radical surgical resection could beneﬁt most but not all patients due to individual variability in lymphatic drainage patterns^7^;^8^ The ideal extent of lymphadenectomy in CRC remains a matter of debate among those favoring standard techniques and the proponents of extensive removal of LNs during surgical treatment.^9^

Indocyanine green (ICG) easily penetrates the lymphatic vessels due to its low molecular weight and hydrophilic properties.^10^ Mapping with ICG can help determine individual lymph flow in each case, and thus perform an adequate resection also in patients who do not have a typical lymphatic flow. On the other hand, there have been reports that ICG maps out normal lymphatics rather than metastatic lymphatics and is less sensitive in lymphatics involved by cancer. This is especially evident when the lymphatics are blocked by malignant cells, altering the normal lymphatic flow.^11^;^12^

In our review, we adopted a critical perspective on using ICG fluorescence imaging (ICG-FI) as a technique to individualize the identification of LNs and lymphatic pathways. By enhancing lymphatic drainage identification, ICG-FI holds promise in improving the precision of surgical lymphadenectomy, lateral pelvic LN dissection (LPLND), and sentinel LN (SLN) detection. These efforts may ultimately result in a precise surgical approach that would allow tailoring the extent of surgery to the individual variability of cancer spread.^13^;^14^ However, the main question remains whether this would authentically impact patient outcomes and overall survival (OS).

## AIM

This scoping review aimed to evaluate the current evidence on the role of ICG fluorescence lymphatic mapping in CRC surgery, assess its impact on surgical outcomes, and identify priorities for future research to optimize its clinical application.

## MATERIALS AND METHODS

We conducted a scoping review of the English-language literature of the MEDLINE database via PubMed, encompassing publications from January 2005 through January 2025. The checklist of Preferred Reporting Items for Systematic reviews and Meta-Analyses extension for Scoping Reviews (PRISMA-ScR) was followed.^15^

### Inclusion and exclusion criteria

The inclusion criteria were as follows: 1) studies investigating ICG fluorescence lymphatic mapping, including technical aspects; 2) studies involving adult populations with CRC; and 3) peer-reviewed original articles (quantitative, qualitative, or mixed-methods). The exclusion criteria comprised: 1) nonclinical studies (eg, animal / cadaveric experiments, in vitro models, computational simulations without clinical validation); 2) studies focusing on nonlymphatic ICG applications (eg, tumor perfusion, angiography) or lacking relevance to CRC surgery; and 3) nonoriginal research (eg, conference abstracts, editorials).

### Data extraction and analysis

The search strategies were drafted by one of the authors (SS) and further refined through team discussion. The final search strategy for MEDLINE search can be found in Table 1 The search results were exported into Mendeley Reference Manager (Elsevier, London, United Kingdom), and duplicates were removed automatically based on digital object identifiers. To ensure the uniqueness of the included data, we manually selected only the most recent publications when singling out articles from the same research group with overlapping study periods. The electronic database search was supplemented by exploring the ICG manufacturer website,^16^ surgical professional organizations’ profiles,^17^;^18^;^19^ and citation scanning of relevant reviews.

**Table 1:** MEDLINE search strategy**^a^**

Searh	Query
1	“Indocyanine green” [MeSH Terms] OR “ICG” [All Fields] OR “ICG fluorescence” [MeSH Terms]
2	“Lymphatic vessels” [MeSH Terms] OR “lymphatic mapping” [All Fields] OR “fluorescence lymphatic mapping” [All Fields]
3	“Colorectal neoplasms” [MeSH Terms] OR “colorectal cancer” [All Fields] OR “colorectal” [All Fields] OR “colon cancer” [All Fields] OR “rectal cancer” [All Fields]
4	“Surgery” [MeSH Subheading] OR “surgery” [All Fields] OR “surgical” [All Fields]
–	1, 2, 3, AND 4
–	Only human study

**a **Literature search was performed on February 14, 2025.

Abbreviations: ICG, indocyanine green; MeSH, Medical Subject Headings

To ensure consistency, 2 reviewers (SS and YR) initially screened the same publications, discussed their findings, and refined the screening and data extraction manually before proceeding with the full review. Two authors (SS and MR) independently conducted the initial screening of titles and abstracts using Rayaan software (Rayyan Systems, Inc., Cambridge, Massachusetts, United States). Both reviewers then screened the full texts of all publications identified as potentially relevant. Any disagreements regarding study selection and data extraction were resolved through consensus. The reviewers jointly developed a data-charting form, extracted the data, and refined the form iteratively through discussion.

We collected the information on the article publication details (eg, author, publication year, journal name, funding sources) and methodology (eg, protocol use, inclusion criteria, literature search approach, screening, and data collection process).

A qualitative analysis of the collected articles was conducted to identify the main themes, trends, and gaps in research regarding the clinical role of ICG fluorescence lymphatic flow mapping in CRC surgery. The analysis involved identifying and categorizing recurring themes, analyzing the authors’ narratives and arguments, and searching for patterns and trends in the scientific literature.

## RESULTS

**Figure 1:**
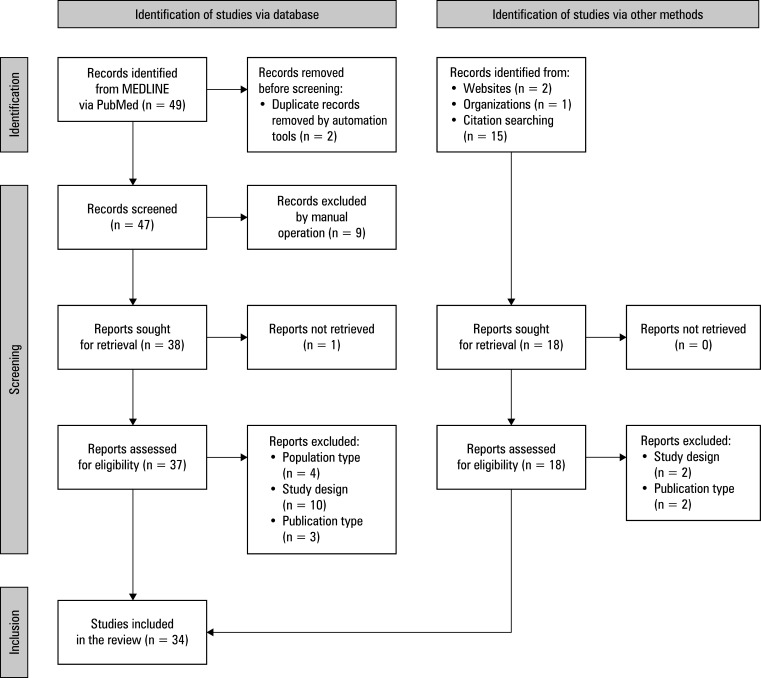
Preferred Reporting Items for Systematic reviews and Meta-Analyses flow diagram of the selection of studies

A total of 67 citations were identified from searches of the electronic database and review of other sources. Based on the title and the abstract, 9 records were excluded, and 1 could not be retrieved. Fifty-five full-text articles were retrieved and assessed for eligibility. Of these, 21 were excluded for the following reasons: 4 included a different patient population, 12 had inappropriate study designs, and 5 were considered ineligible publication types (eg, video correspondence, case reports, commentaries). The remaining 34 studies were considered eligible. The PRISMA flow diagram of this process is shown in Figure 1.

All 34 articles included in the study were assigned to predefined categories. Some articles were placed in multiple groups if their research addressed various aspects of ICG utilization. The following subtopics were created for qualitative analysis: 1) mesenteric lymphatic mapping; 2) SLN assessment; and 3) LPLND. The study characteristics are listed in Table 2, and the main outcomes are collected in Table 3.

**Table 2:** General information about the included studies

Study	Year	Predefined categories			Patients, n	Design	Tumor Location
		MLM	SLN	LPLND			
^20^	2025	x	–	–	129	Prospective	Rectum
^21^	2024	x	x	–	1552	Review	Colorectal area
^22^	2024	x	x	–	462	Review	Colorectal area
^44^	2024	–	–	x	150	Retrtospective	Rectum
^23^	2024	x	x	–	151	Retrtospective	Colorectal area
^54^	2024	–	–	x	108	Prospective	Rectum
^24^	2024	x	–	–	62	Prospective	Colon
^25^	2024	x	–	–	921	Retrtospective	Right colon
^26^	2023	x	–	–	–	Review	Colorectal area
^27^	2023	x	–	x	–	Review	Colorectal area
^28^	2023	x	x	–	146	Prospective	Colorectal area
^29^	2023	x	–	–	–	Review	Colorectal area
^30^	2023	x	–	–	203	Prospective	Right colon
^46^	2023	–	x	–	136	Prospective	Colon
^55^	2023	–	–	x	172	Retrospective	Rectum
^31^	2022	x	–	–	77	Prospective	Colorectal area
^32^	2022	x	–	–	21	Prospective	Colorectal area
^33^	2022	x	–	–	192	Prospective	Colorectal area
^34^	2020	x	x	–	–	Review	Colorectal area
^35^	2020	x	–	–	57	Prospective	Colon
^43^	2020	–	x	–	95	Prospective	Colorectal area
^36^	2020	x	–	–	–	Review	Colorectal area
^37^	2020	x	–	–	106	Retrtospective	Right colon
^51^	2019	–	x	–	30	Review	Colon
^52^	2019	–	x	–	10	Prospective	Colon
^38^	2018	x	x	–	10	Prospective	Colon
^39^	2018	x	–	–	16	Prospective	Colorectal area
^50^	2017	–	x	–	30	Prospective	Colon
^49^	2016	–	x	–	5	Prospective	Rectum
^40^	2016	x	–	–	21	Prospective	Colorectal area
^41^	2012	x	x	–	18	Prospective	Colorectal area
^48^	2012	–	x	–	26	Prospective	Colon
^42^	2011	x	–	–	–	Review	Colon
^47^	2008	–	x	–	26	Prospective	Colon

Abbreviations: LN, lymph node; LPLND, lateral pelvic lymph node dissection; MLM, microcystic lymphatic malformation

**Table 3:** Main outcomes of the included studies

Study	Year	Group	Complicationrate, %	P value	Number of harvested LNs				Aberrant LNs				Postoperative hospital stay, d	P value	OS, %	P value	LRR, %	P value
					Total	P value	Metastatic	P value	Total	P value	Metastatic	P value						
^20^	2025	ICG-FI	–	0.52	median, 20 (range, 5–57)	0.41	0 (0–25)	0.53	–	–	–	–	median, 6 (range, 3–11)	<⁠0.001	–	–	–	–
		Non-ICG			median, 17 (range, 4–47)		1 (0–20)						median, 8 (range, 4–33)					
^21^	2024	ICG-FI	20.9	0.39	weighted mean, 23.5	<⁠0.001	61/218 (28%)	0.08	–	–	–	–	–	–	94.1	0.61	7.6	0.38
		Non-ICG	24.5		weighted mean, 18.9		96/333 (28.8%)								93.1		9.7	
^22^	2024	Review	–	–	–	–	–	–	–	–	–	–	–	–	–	–	–	–
^44^	2024	ICG-FI	27.8	0.18	–	–	–	–	5.1%	–		–	mean (SD), 16.5 (2.7)	0.06	90.3	>0.05	12.6	0.59
		Non-ICG	38						–		–		mean (SD), 17.1 (5.9)		91.6		9.8	
^23^	2024	ICG-FI	60	0.21	median, 39 (range, 14–78)	0.001	median, 1 (range, 0–23)	0.55	–	–	–	–	median, 12 (range, 8–14)	0.06	–	–	–	–
		Non-ICG	43.4		29 (11–70)		median, 2 (range 0–26)						median, 9 (range, 7–13)					
^54^	2024	ICG-FI	3.3	0.62	median, 29 (range 18–37)	0.66	–	–	median, 12 (range, 8–19)^a^	0.01	median, 1 (range, 1–2)	0.439	median, 5 (range, 5–6)	0.15	–	–	–	–
		Non-ICG	10		median, 27 (range, 21–37)				median, 9 (range, 6–13)^a^		median, 1 (range, 1–2)		median, 6 (range, 5–8)					
^24^	2024	ICG-FI	–	–	28^c^	0.78	–	–	20.9%	–	–	–	–	–	–	–	–	–
		Non-ICG			30^c^				–		–							
^25^	2024	ICG-FI	38	0.31	median, 31 (23–39)	0.047	27.3%	>0.99	–	–	–	–	median, 7 (range, 5–8)	0.35	94.5	0.3	2	0.25
^26^	2023	Review	–	–	–	–	–	–	–	–	–	–	–	–	–	–	–	–
^27^	2023	Review	–	–	–	–	–	–	–	–	–	–	–	–	–	–	–	–
^28^	2023	ICG-FI	–	–	–	–	–	–	n = 72	–	45.8%	–	–	–	–	–	–	–
^29^	2023	Review	–	–	–	–	–	–	–	–	–	–	–	–	–	–	–	–
^30^	2023	ICG-FI	–	–	42^c^	0.5	1^c^	0.49	–	–	–	–	–	–	–	–	–	–
		Non-ICG			39^c^		1.5^c^											
^46^	2023	ICG-FI	–	–	mean (SD), 20.2 (10.6)	–	–	–	2.22%	–		–	–	–	–	–	–	–
^55^	2023	ICG-FI	–	–	median, 14 (range, 10–18)	<⁠0.001	mean (SD), 9 (15.5)	0.79	–	–	–	–	–	–	93.1	0.2	0^b^	0.05
		Non-ICG			median, 9 (range, 5–11)		mean (SD), 7 (12.1)								85.9		9.3^b^	
^31^	2022	ICG-FI	20	–	median, 16 (range, 5–24)	–	–	–	41.4%	–		–	median, 4 (range, 3–16)	–	–	–	–	–
^32^	2022	ICG-FI	–	–	–	–	–	–	19%	–		–	–	–	–	–	–	–
^33^	2022	ICG-FI	–	–	median, 33.3 (range, 7–231)	<⁠0.001	median, 4.2 (range, 1–22)	0.86	–	–	–	–	–	–	–	–	–	–
		Non-ICG			median, 22.8 (range, 2–72)		median, 4.2 (range, 1–14)											
^34^	2020	Review	–	–	–	–	–	–	–	–	–	–	–	–	–	–	–	–
^35^	2020	ICG-FI	–	–	n = 222	–	n = 12	–	–	–	–	–	–	–	–	–	–	–
		Non-ICG			n = 1203		n = 56											
^43^	2020	ICG-FI		–	–	–	–	–	–	–	–	–	–	–	–	–	–	–
^36^	2020	Review	–	–	–	–	–	–	–	–	–	–	–	–	–	–	–	–
^37^	2020	ICG-FI	4	0.37	median, 41 (range, 23–69)	0.02	median, 0 (range, 0–7)	0.28	32%	–		–	median, 7 (range, 5–10)	0.3	–	–	–	–
		Non-ICG	10		median, 30 (range, 144–76)		median, 0 (range, 0–12)		–		–		median, 6 (range, 4–24)					
^51^	2019	Review	–	–	–	–	–	–	–	–	–	–	–	–	–	–	–	–
^52^	2019	ICG-FI	–	–	n = 102	–	n = 1	–	–	–	–	–	–	–	–	–	–	–
^38^	2018	ICG-FI	–	–	median, 22 (range, 14–49)	–	median, 2 (range, 0–8)	–	20%	–	100%	–	–	–	–	–	–	–
^39^	2018	ICG-FI	–	–	n = 287	–	n = 17	–	–	–	–	–	–	–	–	–	–	–
^50^	2017	ICG-FI	–	–	median, 34 (range, 27–39)	–	–	–		–		–	–	–	–	–	–	–
^49^	2016	ICG-FI	20	–	–	–	–	–	–	–	–	–	–	–	–	–	–	–
^40^	2016	ICG-FI		–	–	–	–	–	23.5%	–	–	–	–	–	–	–	–	–
^41^	2012	ICG-FI		–	–	–	–	–	22%	–		–	–	–	–	–	–	–
^48^	2012	ICG-FI		–	median, 32.9 (range, 10–143)	–	–	–	–	–	–	–	–	–	–	–	–	–
^42^	2011	Review	–	–	–	–	–	–	–	–	–	–	–	–	–	–	–	–
^47^	2018	ICG-FI		–	mean (SD), 13.5 (8.9)	–	–	–	–	–	–	–	–	–	–	–	–	–

**a **Lateral lymph nodes

**b **Lateral local recurrence

**c **Values are reported as mean values without SD (as presented in the original article)

Abbreviations: FI, fluorescence imaging; LRR, local recurrence rate; OS, overall survival; others, see Table 1

### Mesenteric lymphatic mapping

Twenty-three evaluated studies ^20^;^21^;^22^;^23^;^24^;^25^;^26^;^27^;^28^;^29^;^30^;^31^;^32^;^33^;^34^;^35^;^36^;^37^;^38^;^39^;^40^;^41^;^42^ investigated using ICG for mesenteric lymphatic mapping, examining its association with precision in mesenteric LN dissection. Since 2023, the European Association for Endoscopic Surgery consensus statement^26^ on ICG fluorescence-guided surgery has suggested that ICG lymphatic mapping is safe and feasible for identifying lymphatic anatomy during colorectal procedures. However, the clinical value of ICG mapping has yet to be fully defined.

#### Indocyanine green administration and early postoperative outcomes

The dosage and timing of peritumor injection of the ICG solution for LNs and lymph flow mapping vary across studies. The injection was typically performed either submucosally during intra- or preoperative (up to 24 h before) colonoscopy or subserosally during primary surgery.^29^;^31^;^32^ The ICG solution (typically 0.25 mg/ml, diluted in sterile water) was injected at 1–3 sites near the tumor’s distal margin (0.5 ml per injection point). Injections at multiple sites had a higher successful mapping rate than a single injection (75.9% vs 38.5%, respectively; *P* <⁠0.001).^33^ No studies reported significant adverse events associated with an ICG injection. Isolated cases of technical failure, such as ICG extravasation and spillage, have been observed.^22^;^24^;^30^;^32^;^37^ A number of recent studies suggest that high body mass index (BMI) may hinder the visualization of lymphatic channels and nodes with ICG due to increased tissue thickness and decreased signal penetration.^35^;^43^;^33^ showed that nonobese status (BMI <⁠25 kg/m²) is related to the success of ICG-FI (*P* = 0.002). In contrast, ^35^ found no differences in the rate of visualized lymphatic flow between patients with higher BMI (≥22.9 kg/m^2^) and lower BMI (<⁠22.9 kg/m²; *P* = 0.49).

No studies reported differences in total postoperative complications between ICG-FI mapping and control groups. However, particular complication types varied between the groups in some studies. ^23^ who evaluated ICG-guided laparoscopic para-aortic lymphadenectomy in left-sided CRC, observed a notably higher rate of chylous leakage (35% vs 11.3%; *P* = 0.04) and more grade II (Clavien–Dindo) complications (60% vs 28.3%; *P* = 0.01) in the ICG group than the controls. In contrast, ^44^ reported that the frequency of urinary dysfunction was lower in the ICG group than in the control group (*P* = 0.04). Moreover, only 1 study, performed by ^20^ showed that the ICG-FI mapping group with rectal cancer surgery had a shorter postoperative hospital stay (6 vs 8 d; *P* <⁠0.001), as compared with the control cohort. None of the studies reported a difference in the duration of surgery, rehospitalizations, or mortality within 30 days after surgery.

#### Quality of lymphadenectomy using indocyanine green lymphatic mapping

A meta-analysis ^21^ of 7 studies showed that the ICG group, as compared with the conventional laparoscopy group, demonstrated a greater number of harvested LNs (23.5 vs 18.9; *P* <⁠0.001), and the metastatic rate in ICG-positive LNs ranged from 1.1% to 81.8% across the studies. The lymph flow detection rate ranged from 75.4% to 100%, with an overall pooled rate of 89.9%. Interestingly, ^35^ found that the rate of visualized lymphatic flow was higher in patients with a lower (Clavien–Dindo grade 0–II) than higher (Clavien–Dindo grade III and IV) clinical stage (*P* = 0.01). A meta-analysis of 11 studies by ^34^ demonstrated poor overall performance of ICG in detecting metastatic LNs (sensitivity, 64.3%; specificity, 65%).

^20^ prospectively analyzed LN stations 251–253 (Japanese Society for Cancer of the Colon and Rectum classification) in 129 rectal cancer patients undergoing laparoscopic total mesorectal ecxision with D3 lymphadenectomy, comparing the ICG-FI mapping group with the controls. Station 251 included perirectal and superior rectal artery (SRA) nodes up to its origin; station 252 comprised inferior mesenteric artery (IMA) nodes between the left colic artery (LCA) and SRA branch; and station 253 covered IMA nodes from the aortic origin to the LCA. The ICG-FI group showed a higher median yield of station 253 LNs (2 vs 1; *P* = 0.007), with no differences in stations 251 and 252. No increase in metastatic positive LNs in station 253 was observed in the ICG-FI group. Furthermore, ^33^ found that the number of D3 LNs during CRC surgery was higher in the ICG-FI mapping group than in the control group (*p* <⁠0.001). However, the number of metastatic the D3 LNs was not related to the success of ICG-FI mapping (*P* = 0.94). Similar findings were presented by ^37^ in patients with advanced right-sided colon cancer. The use of ICG-FI was an independent factor for retrieving a greater number of overall (39 vs 30; *P* = 0.003) and central LNs (14 vs 7; *p* <⁠0.001), but the number of metastatic LNs was similar in the 2 groups. In contrast, ^24^ showed that the total number of harvested LNs and central LNs in patients with colon cancer in the ICG group was not different from those in the non-ICG group (28 vs 30; *P* = 0.78 and 8 vs 8, respectively; *P* = 0.86).

#### Detection of aberrant lymph nodes

Aberrant drainage is lymphatic drainage to LNs outside the standard resection margin, requiring a change in the extent of operation.^45^ Ten studies reported detection of aberrant LNs using ICG-FI mapping.^24^;^28^;^31^;^32^;^37^;^38^;^40^;^41^;^44^;^46^ The aberrant LN detection frequency varied across the studies, ranging from 2.2% to 50%. In 8 studies, metastases were not detected in aberrant LNs. However, ^28^ analyzed data of 146 CRC patients in the European registry on fluorescence image-guided surgery. They found that ICG identified LNs outside standard lymphatic stations in 50% of the cases, with 45.8% of the retrieved LNs being metastatic. Additionally, ^38^ showed that in 20% of the patients (n = 2), additional LNs outside the proposed colon cancer resection margins were detected, and these were metastatic in both patients.

#### Indocyanine green lymphatic mapping and lymphadenectomy impact the long-term survival rate

While the impact of ICG-FI lymphatic mapping on long-term oncologic outcomes remains inconclusive, the first meta-analysis^21^ or separate studies^25^;^44^ have begun to address this question. ^21^ conducted a meta-analysis of 18 studies, but only 2 of them compared the estimated 3-year treatment outcomes. The authors found that ICG-FI mapping did not improve the OS rate (94.1% vs 93.1%; *P* = 0.61), relapse-free survival rate (85.1% vs 86.2%; *P* = 0.72), or local recurrence rate (7.6% vs 9.7%; *P* = 0.38), as compared with conventional laparoscopic surgery.

### Sentinel lymph node assessment

SLN mapping is a diagnostic procedure to identify the first LNs to which a tumor drains. Fourteen studies^21^;^22^;^28^;^34^;^38^;^41^;^43^;^46^;^47^;^48^;^49^;^50^;^51^ investigated the feasibility, safety, and effectiveness of ICG-FI in identifying SLNs and detecting the potential for using this technique in CRC patients.

ICG-FI can accurately identify SLNs in CRC, with detection rates ranging from 75.5% to 100%.^21^;^28^;^34^;^38^;^41^;^43^;^47^;^50^;^28^ found that ICG-FI SLN navigation was positive in 75.5% of the cases, with a metastatic rate of 14.7% in the retrieved SLNs. ^53^ found that for SLNs, the procedure obtained sensitivity and negative predictive value of 73% and 96.2%, respectively. In a meta-analysis conducted by ^51^ the sensitivity of ICG-FI for identifying SLN metastases in colon cancer was 63%, and the negative predictive value was 81%.

Another retrospective study conducted by ^44^ evaluated the prognostic impact of ICG-guided lateral SLN biopsy (SLNB) on oncologic outcomes in patients with clinical stage II/III lower rectal cancer without suspected lateral LN metastasis, comparing it to prophylactic LPLND. The study found that ICG-guided SLNB and prophylactic LPLND had similar long-term oncologic outcomes, including the cumulative incidence of local recurrence (*P* = 0.59), lateral local recurrence (LLR) rate (*P* = 0.91), cancer-specific survival (*P* = 0.61), OS (*P* = 0.93), recurrence-free survival (*P* = 0.89), local recurrence-free survival (*P* = 0.41), and distant recurrence-free survival (*P* = 0.93).^44^

Scientists attempt to improve the ICG-FI SLN technique for CRC by combining it with other methods, such as hybrid imaging or ultrastaging.^46^;^48^;^51^ Ultrastaging means using more sensitive histopathology techniques, such as immunohistochemistry (IHC) or additional assessment, to detect micrometastases in SLNs. A study by ^46^ found a 10% upstaging rate (3/30 patients), demonstrating that ICG-guided SLN mapping with ultrastaging via IHC detects metastases, particularly in T1/T2 colon cancer, that may be missed on conventional hematoxylin and eosin staining, enabling adjuvant therapy. Moreover, ^39^ assessed the feasibility of combining ICG-FI with intraoperative 1-step nucleic acid amplification (OSNA) for SLN assessment. Of the 9 ICG-positive LNs identified intraoperatively, only 1 (11.1%) was OSNA-positive for metastasis. ICG-guided nodes correctly reflected the final nodal status (N0/N1) in 3 out of 6 patients (50%). In 3 cases, ICG-identified nodes were negative despite N1/N2 disease, resulting in a 50% discordance.^39^

On the other hand, ^52^ investigated combining ICG with positron emission tomography / computed tomography (PET/CT) lymphoscintigraphy using submucosal [^89^Zr]Zr-Nanocoll to enhance SLN detection in early-stage colon cancer. Intraoperatively, ICG identified 21 of 27 SLNs (78%), all showing fluorescence and radioactivity. However, 6 SLNs within 2 cm of the tumor were missed due to the ICG’s shine-through effect. At the same time, PET/CT detected all 27 SLNs preoperatively.^52^.

### Lateral pelvic lymph node dissection

Four studies^27^;^44^;^54^;^55^ used ICG to guide LPLND. LPLND in CRC involves the removal of LNs along the internal iliac vessels and their branches, including the obturator, internal pudendal, inferior gluteal, and superior gluteal LNs. While Japanese guidelines^56^ routinely recommend LPLND for lower rectal cancers in tumors invading beyond the muscularis propria, Western (National Comprehensive Cancer Network / European Society for Medical Oncology) guidelines^57^;^58^ reserve LPLND only for magnetic resonance imaging / PET-confirmed metastases without neoadjuvant therapy response.

The efficacy of ICG-FI lymph mapping in combination with real-time inferior vesical artery (IVA) angiography during laparoscopic LPLND for lower rectal cancer was investigated by ^54^ They found that the ICG group had a higher number of IVA preservation ratio (93.1% vs 56%; *p* <⁠0.001) and a greater number of harvested LNs (12 vs 9; *P* = 0.01) than the non-ICG group. However, the number of positive LNs was the same in both groups (1 vs 1; *P* = 0.44).

^55^ demonstrated that ICG-FI–guided laparoscopic LPLND reduced the LLR rate in middle-lower rectal cancer patients, with a 3-year cumulative LLR of 0% in the ICG-FI group vs 9.3% in the conventional LPLND group (*P* = 0.05).

## DISCUSSION

The most established application of ICG-FI in CRC surgery is the assessment of bowel perfusion to prevent anastomotic leakage.^26^;^59^;^61^ A recent randomized controlled trial (RCT) confirmed its safety and potential to reduce leakage risk in laparoscopic rectal resection.^61^ However, additional intraoperative uses have been proposed, including lymphatic mapping, ureter visualization, and identification of nerve plexuses.^13^;^29^;^36^;^62^ These alternative applications remain underexplored, with limited supporting evidence and a lack of high-quality RCTs. Given the potential role of lymphatic mapping in improving oncologic outcomes, our review focused on this specific use of ICG-FI. We identified and analyzed 34 studies published between 2005 and 2025 investigating ICG-guided visualization of lymphatic pathways in CRC.

### Mesenteric lymphatic mapping

Our review suggests that, while promising, current evidence remains insufficient to support the routine use of ICG-FI lymphatic mapping for improving oncologic outcomes in CRC. The understanding of lymphatic drainage patterns and nodal metastases in CRC is still evolving, and a universally accepted anatomical model has yet to be established.^9^;^11^;^45^ This is highlighted by multiple studies reporting the presence of aberrant LNs.^24^;^28^;^31^;^32^;^37^;^40^;^41^;^46^;^63^ Despite these uncertainties, ICG-guided mapping continues to attract research interest, particularly for selected patient subgroups, such as individuals who have undergone neoadjuvant therapy or prior colorectal surgery, or those presenting with inconclusive preoperative imaging.^23^;^44^;^54^ A key unresolved issue is whether the methodologies used in current studies are optimal, or if robust, multicenter RCTs are needed to definitively evaluate the clinical utility of lymphatic mapping in CRC surgery.

### Sentinel lymph node assessment

The concept of SLN mapping in colon cancer has been explored for decades, but has not gained the same clinical relevance as in breast cancer, melanoma, or gastric cancer.^14^;^64^;^65^;^66^ The limited accuracy reported in earlier studies may reflect the inclusion of patients with advanced disease or those treated with limited or minimally-extensive surgical resections. However, the growing adoption of organ-preserving strategies in CRC treatment, such as total neoadjuvant therapy and watch-and-wait protocols, highlights the need for minimally-invasive methods of nodal staging.^67^;^68^ ICG-guided lymphatic mapping could serve as a precision diagnostic tool, allowing targeted nodal biopsy to support individualized treatment planning.^43^;^46^ Compared with traditional SLN detection methods involving blue dye, radiocolloids, or their combination, ICG-FI offers several advantages.^36^;^52^ Its higher fluorescence intensity and deeper tissue penetration enable superior real-time visualization without the need for ionizing radiation. Additionally, the risk of adverse reactions, including anaphylaxis, is extremely low.^48^

### Lateral pelvic lymph node dissection

Surgical approaches to LPLND in patients with CRC vary internationally and are influenced by institutional treatment concepts.^69^ This procedure is technically challenging and associated with significant risks, including intraoperative hemorrhage and postoperative neurological deficits.^70^ Nevertheless, in carefully selected patients, such as younger individuals or those with suboptimal response to neoadjuvant chemotherapy, LPLND may be clinically justified despite its invasiveness. Although high-quality data are limited, the findings of this review suggest that ICG-FI may enhance the safety and precision of LPLND. In the included studies, ICG-FI was associated with improved anatomical identification, higher preservation rates of critical vascular structures (eg, IVA), and increased LN yield. Moreover, 1 study indicated a potential reduction in LLR with ICG-FI guidance.^55^ These preliminary findings support the hypothesis that intraoperative ICG lymphatic mapping could mitigate some of the inherent risks of LPLND while potentially improving oncologic outcomes.

This review has several limitations. Not all available databases were included in the search, and the cost-effectiveness of ICG-FI was not specifically analyzed. Furthermore, issues related to diversity, equity, and inclusion were not addressed due to the unavailability of the reviewed literature.

## CONCLUSIONS

ICG-FI enhances intraoperative lymphatic visualization in CRC surgery and may increase the precision of lymphadenectomy. While it facilitates the detection of atypical drainage pathways and sentinel nodes, current evidence does not support a clear survival benefit. Further standardization and high-quality studies are needed before routine implementation.
